# Editorial for Special Issue Foodborne Pathogens: Infections and Pathogenesis

**DOI:** 10.3390/microorganisms11061544

**Published:** 2023-06-10

**Authors:** Mónica Oleastro, Ana Botelho

**Affiliations:** 1 Infectious Diseases Department, National Institute of Health Dr. Ricardo Jorge, 1649-016 Lisbon, Portugal; 2 National Reference Laboratory for Animal Health, National Institute for Agrarian and Veterinary Research, 2780-157 Oeiras, Portugal

Foodborne microorganisms substantially impact food safety and contribute considerably to the public health and economic burden of infectious diseases worldwide.

In the Special Issue on “Foodborne Pathogens: Infections and Pathogenesis”, seven research papers and three reviews were published, between February 2022 and January 2023. Studies addressing *Campylobacter* spp, *Campylobacter jejuni* and/or *Listeria monocytogenes* accounted for seven out of the ten papers that comprise this Special Issue, showcasing their relevance as agents of foodborne infections and outbreaks, which, according to the EFSA, accounted for 257 and 23 outbreaks in 2021, respectively (EFSA https://www.efsa.europa.eu/en/microstrategy/FBO-dashboard (accessed on 22 May 2023)).

The two review papers on *C. jejuni* provided current knowledge on the pathogenic profile of this agent and the approaches to control human campylobacteriosis, which is strongly associated with poultry, beef, and pork consumption [1]. Interdisciplinary approaches are essential to understand the biology of this agent and to control outbreaks, namely the use of mathematical metabolic models [2].

Tolerance and adaptation to stress conditions are features that contribute to the survival of *L. monocytogenes* in different niches and its’ infectious capability. The regulation of stress response genes overlaps with the regulation of virulence genes, facilitating its transition from a saprophytic survival state to a pathogenic state once inside the host. Knowledge of these pathways is crucial for identifying potential targets to control foodborne diseases caused by *L. monocytogenes*. These topics were addressed in one review paper [3].

Most foodborne diseases have a zoonotic origin; therefore, an integrated approach from farm to fork must be adopted in food safety to protect consumers. Apart from the economic losses for farmers, abortion in livestock also raises public health concerns. One paper reported lamb mortality on flocks due to the co-infection of *L. monocytogenes* and *Toxoplasma gondii*, while the source attribution is not always easy to determine [4].

Whole genome sequencing (WGS) analysis is a powerful tool that can reveal relevant information regarding genetic traits, such as virulence and resistance genes, genetic diversity, and relationships between strains, assisting in source attribution. Applying WGS to *L. monocytogenes* serogroup IIa isolates, from food and food production environment, revealed a high prevalence of disinfectant’s resistance genes, and genes coding for virulence factors and biofilm formation. The comparison of molecular profiles of other previous isolates suggests that some *L monocytogenes* strains have the ability to persist in food production environments for a long time, constituting a potentially worrisome and recurrent source of infection for humans [5].

Routine surveillance and outbreak investigation of foodborne diseases is ever more critical, due to the tendency to return to traditional food production using raw materials and, eventually, poor hygienic practices. In a study performed on traditional cured raw milk Portuguese cheese, the pathogenic agents *L. monocytogenes*, coagulase-positive *Staphylococci* and *Escherichia coli*, were detected in 15.6%, 16.9% and 10.1% of the samples, respectively. Therefore, these food production setups require effective prevention and control measures associated with multi-sectoral WGS data integration [6].

Other traditional habits, such as hunting and consuming of related products, namely in Mediterranean countries, can create an emerging public health concern. In fact, it has been shown that the *Hepatitis E* virus can survive across the years, mainly in wild boars’ reservoirs, and can predictably be transmitted to humans via the oral-fecal route [7].

The ability to isolate a pathogenic agent is a prerequisite in many characterization and performance research studies, as it allows for a comprehensive approach to the source of infection and transmission routes of foodborne outbreaks. A new solid chromogenic medium (CAMPYAIR) with antibiotic supplements, that supports the growth of *Campylobacter* isolates within 48 h of incubation in aerobic atmospheres, was developed. This new selective, differential medium could help reduce the costs, equipment, and technical demand required for *Campylobacter* isolation from clinical and environmental samples [8].

The onset of pathogenicity and virulence shifts is a complex and multifactorial issue depending, namely, on the environment, mechanisms of modulation, and interaction between the host and the infectious agent. All these factors should be evaluated separately to reach robust conclusions. Therefore, the influence of oxygen levels in the modulation of virulence and pathogenicity-associated traits of *Arcobacter butzleri* was studied, and it was demonstrated for the first time that oxygen levels can modulate the pathogenicity of *A. butzleri* [9].

Regulatory molecules, such as small non-coding RNAs can also control virulence since they are critical for quorum-sensing pathways and the regulation of some virulence factors. As such, a comprehensive transcriptome profile of *Vibrio parahaemolyticus* during infection in Caco-2 cells, has been released [10].

The research and reviews conducted in the ten papers published in this Special Issue contribute substantially to gathering knowledge on the infection and pathogenesis of foodborne pathogens.

At the time of this writing, the papers published in this Special Issue were viewed 15,148 and cited 22 times based on statistics from Microorganisms website.
